# In Situ Measurement of Spindle Radial Error for Ultra-Precision Machining Based on Three-Point Method

**DOI:** 10.3390/mi14030653

**Published:** 2023-03-14

**Authors:** Hanwei Xu, Zizhou Sun, Yifan Dai, Chaoliang Guan, Hao Hu, Yu Wang

**Affiliations:** 1 College of Intelligent Science and Technology, National University of Defense Technology, Changsha 410073, China; 2 Hunan Key Laboratory of Ultra-Precision Machining Technology, Changsha 410073, China; 3 Laboratory of Science and Technology on Integrated Logistics Support, National University of Defense Technology, Changsha 410073, China

**Keywords:** radial error, ultra-precision spindle, nanometer-scale measurement, three-point method, Donaldson reversal method

## Abstract

The radial error is an important parameter to evaluate the performance of ultra-precision spindles. The three-point method has not yet been well applied in nanometer-scale measurement due to its disadvantages of harmonic suppression and the complicated error separation process. In order to verify that the three-point method can realize the nanometer-scale measurement of the radial error in the machining environment, an in situ measurement and evaluation system is established. Experiments are performed using the system, and a comparative experiment is conducted to verify the accuracy of the system. The average value and standard deviation of the measurement results are 23.096 nm and 0.556 nm, respectively. The in situ measurement result was in good agreement with the Donaldson reversal method using a commercially available spindle analyzer.

## 1. Introduction

Ultra-precision spindles have been widely used in advanced manufacturing and ultra-precision measurement, supporting development in semiconductor electronics, space exploration and other fields [1,2]. Radial error refers to the deviation of the instantaneous axis of the rotating spindle relative to the average axis in the radial direction. Since the spindle drives the tool to rotate, the radial error will directly affect the depth of cut, and then affect the face accuracy of the machined workpiece [3,4,5]. Therefore, the measurement of the radial error is a key link to design a spindle and evaluate the machining performance of the ultra-precision spindles [6]. Focused on the method of ultra-precision spindle radial error measurement, scholars have carried out a significant amount of research. The proposal of error separation technology is a major breakthrough in the field of spindle radial error measurement [7], promoting the development of spindle metrology. Several error separation techniques were then developed, including the multi-point method [7], multi-step method [8,9] and the Donaldson reversal method [10,11]. The multi-step method is rarely used in the field of ultra-precision measurement because of its complex operation steps.

After the three methods above were put forward, most of the scholars’ research was carried out on the basis of these three measurement methods. Compared with the other two methods, the Donaldson reversal method is based on a simpler principle. Cui et al. [12] constructed a nanometer system for measuring the radial error of aerostatic ultra-precision based on the Donaldson reversal method. The effect of the cogging torque of the motor, the angle deviation, artifact eccentricity and spindle axial motion on the measuring accuracy of the spindle were studied. The accuracy of the measurement system and the validity of the Donaldson reversal method were confirmed. Chen et al. [13] measured the uncertainty of the rotary accuracy of an ultra-precision aerostatic spindle based on the Donaldson reversal method. The results showed that the nonlinear error and the mounting error of the capacitive sensor could affect the measurement accuracy. The Lion Precision Spindle Error Analyzer (SEA) was successfully used by Jerzy Józwik et al. [14] to measure the radial error of the spindle of a DMC 635 eco machining center. The SEA is a representative commercial instrument based on the Donaldson reversal method, but it has extremely high requirements on the accuracy of reverse positioning. It is best to use a precision mechanism for reverse, otherwise a large secondary clamping error will be introduced. In short, although the principle of the Donaldson reversal method is simple, the operation requirements are extremely high, and it is not suitable for the ultra-precision measurement of the radial error of the horizontal spindles.

Another method is the multi-point method [15], mainly including the three-point method and the four-point method [16], which only requires one setup to be measured. According to the research of Eric R. Marsh [17,18], the three-point method can realize nanometer-scale measurement, but experimental technical details were not given. Although the measurement operation of the three-point method is relatively simple, harmonic suppression is a shortcoming of the three-point method that cannot be ignored [19,20]. Research found that the influence of harmonic suppression can be reduced or even ignored by selecting the appropriate sensor angle [21]. Gao et al. [22,23] proposed several new multi-probe methods, which can effectively separate the roundness of the measured workpiece from the spindle radial error, thereby avoiding the problem of harmonic suppression. In addition, some scholars have also systematically studied the three-point method [11,24,25], but most of them have only carried out theoretical research and verified it in the laboratory environment. To sum up, many scholars have conducted in-depth research on the three-point method, but there are still few practical applications of the three-point method in ultra-precision machining measurement.

This paper designs an in situ measurement and evaluation system based on the three-point method, which can realize ultra-precise measurement in the machining environment. The system is used to realize the measurement of 20 nm radial error on an ultra-precision lathe, and the roundness error of the measured standard workpiece can be obtained at the same time. The theoretical analysis and technical details of the experiments are given in detail. The advantages of the three-point method over the Donaldson reversal method in the machining environment is illustrated.

## 2. Mathematical Model and Error Separation Technology

### 2.1. Mathematical Model of the Circular Cross-Sectional Profile

The circular cross-sectional profile of shaft parts has a periodic character and can be decomposed into sinusoidal waves of different orders. These sine waves are superimposed in a defined pattern to obtain the cross-sectional profile.

**Figure 1:**
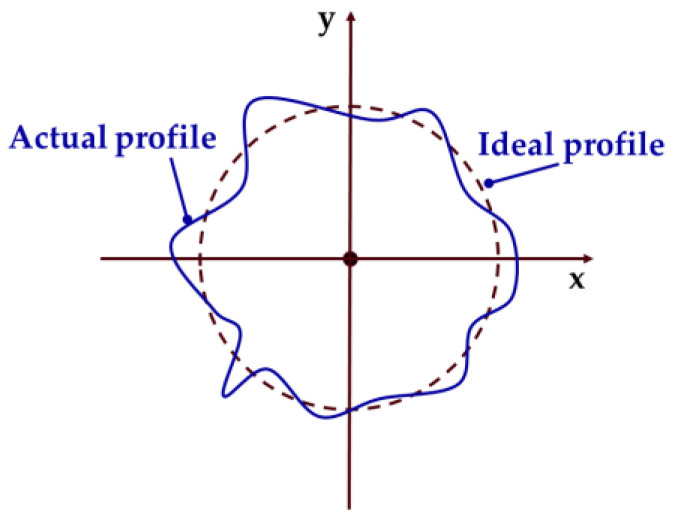
Schematic diagram of one cross-sectional profile.

The circular cross-sectional profile of shaft parts is shown in Figure 1, with the solid line indicating the actual profile and the dashed line indicating the ideal profile. It can be found that the shape of the cross-sectional profile of the shaft parts in rotation will exhibit deterministic periodic signal characteristics, which can be expanded by Fourier series as

(1) $$r(\theta_{p})=r_{0}+\sum_{k=1}^{\infty }\left(a_{k}cosk\theta_{p}+b_{k}sink\theta_{p}\right)$$

where $\theta_{p}$ is the polar angle; $r_{0}$ is the average radius of the circular cross-sectional profile of the workpiece; k is the number of harmonics; and $a_{k}$ and $b_{k}$ are Fourier coefficients of the roundness error profile.

Since the sampling points are discrete in the actual sampling process, the Fourier coefficients can be calculated by Euler’s criterion as

(2) $$a_{k}=\frac{2}{N}\sum_{n=1}^{N}r(n)\cos\frac{2\pi kn}{N}$$

and

(3) $$b_{k}=\frac{2}{N}\sum_{n=1}^{N}r(n)\sin\frac{2\pi kn}{N}$$

where N is the number of sampling points in each circle and n is the n−th of the total number of sampling points in each circle.

**Figure 2:**
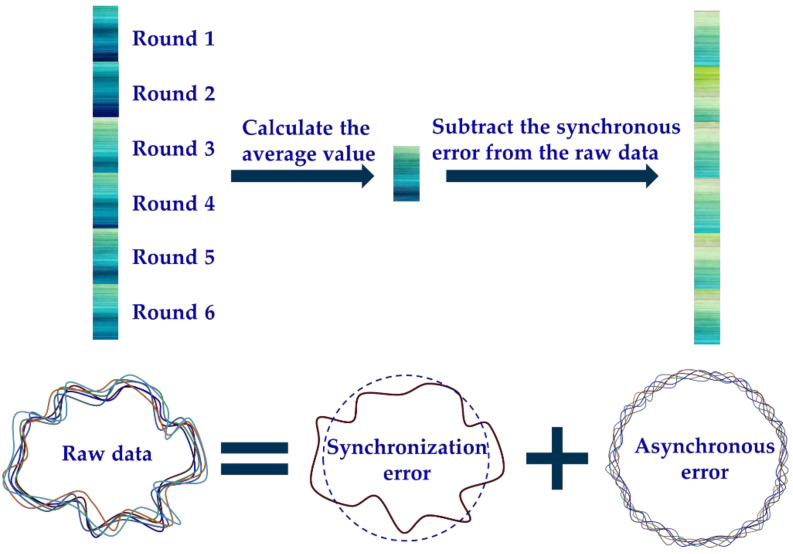
Separation of synchronous and asynchronous errors.

In the actual sampling, a sensor probe with suitable accuracy is selected for data acquisition. The collected data include synchronous error and asynchronous error, and these errors can be separated. The synchronous error can be obtained by averaging the raw data of multiple turns, and the asynchronous error data is obtained by subtracting the synchronous error from the raw data, as shown in Figure 2.

**Figure 3:**
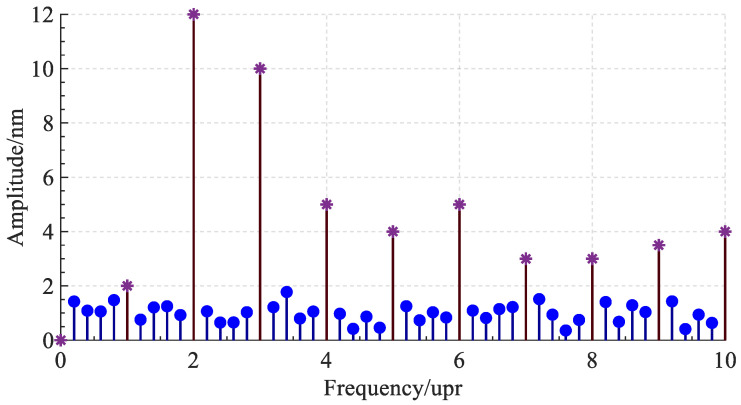
Frequency domain map of original data after Fourier transform.

After Fourier transform, the synchronous error data are distributed in integer multiples of the spindle rotation frequency and its neighborhood, and the asynchronous error data are distributed in non-integer multiples of the rotation frequency interval, as shown in Figure 3. The lines marked with asterisks represent the frequency components of synchronous errors, and the lines with circular marks represent the frequency components of asynchronous errors. The synchronization error data, combined with the data of other probes or other measurement steps, can be further separated into the radial error of the spindle and the roundness error of the standard workpiece. The separation method of the synchronization error will be further introduced below.

### 2.2. Error Separation Techniques

Roundness error separation methods generally include the multi-point method, multi-step method and Donaldson reversal method. Compared with the multi-step method, the three-point method and the reversal method have the advantages of fewer clamping times, thus introducing less error. Therefore, the measurement of radial error of the spindle generally uses the three-point method and the reversal method.

#### 2.2.1. Three-Point Method

**Figure 4:**
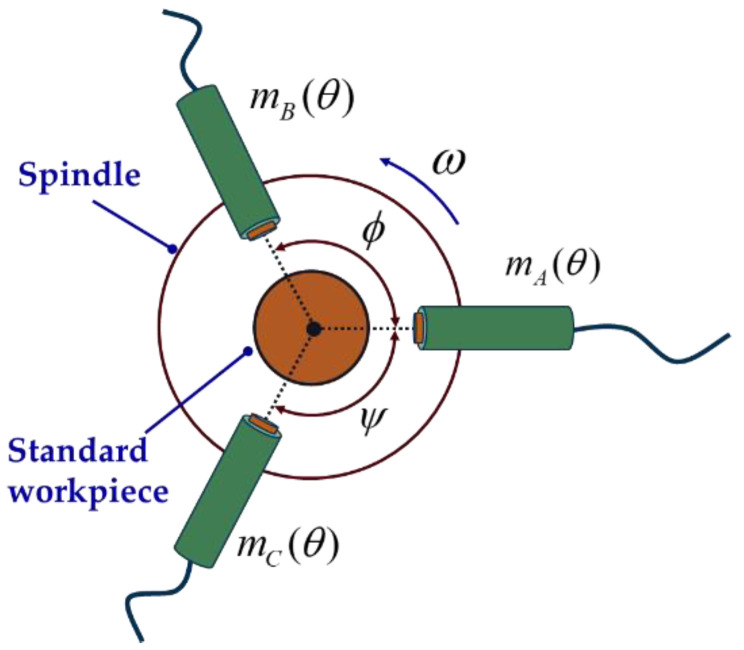
Schematic diagram of sensor distribution of three-point method.

As shown in Figure 4, three sensor probes are placed at a certain angle to collect data synchronously. After removing the asynchronous error and the installation eccentricity of the standard workpiece, the data collected by the three sensors can be expressed as

(4) $$\left\{ \begin{array}{l} m_{A}(\theta )=p(\theta )+x(\theta )\\ m_{B}(\theta )=p(\theta -\alpha )+x(\theta )\cos\alpha +y(\theta )\sin\alpha \\ m_{C}(\theta )=p(\theta +\beta )+x(\theta )\cos\beta -y(\theta )\sin\beta \end{array}\right.$$

where θ is the spindle rotation angle; α is the angle between sensor A and sensor B; β is the angle between sensor A and sensor C; $m_{A}(\theta )$, $m_{B}(\theta )$, $m_{C}(\theta )$ respectively represent the readings of the three sensors after removing the asynchronous error and workpiece eccentricity; p(θ) is the roundness error of the standard workpiece; and x(θ) and y(θ) respectively represent the projected components of the radial error of the spindle on the *x*-axis and *y*-axis.

The radial error of the spindle and roundness error of the standard workpiece can be obtained by analyzing the weighted sum of each sub-formula of Formula (4) using the Fourier transform method.

#### 2.2.2. Donaldson Reversal Method

**Figure 5:**
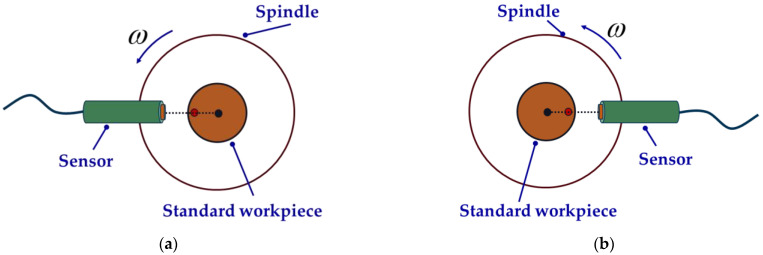
Schematic diagram of Donaldson reversal method measurement: (**a**) before reversal, (**b**) after reversal.

The measurement using the Donaldson reversal method is mainly divided into two steps: first, fix the sensor at the 0° position, and collect the first set of data; then, the measured standard workpiece and the sensor are rotated 180° at the same time to collect the second set of data. The measurement steps of the Donaldson reversal method are shown in Figure 5. Detailed technical details will be introduced in the comparative experiment.

The average value of each point measured before the reversal is $T_{1}$, and that after the reversal is $T_{2}$:

(5) $$\left\{ \begin{array}{l} T_{1}(\theta_{j})=\frac{1}{m}\;\sum_{1}^{m}C_{ij},i=1,2,\cdots ,m;j=1,2,\cdots ,n\\ T_{2}(\theta_{j})=\frac{1}{m}\;\sum_{1}^{m}D_{ij},i=1,2,\cdots ,m;j=1,2,\cdots ,n \end{array}\right.$$

where $C_{ij}$ is the sample value of the j−th point in the i−th cycle before the reversal, and $D_{ij}$ is that after the reversal; i is the number of revolutions sampled; j is the number of sampling points per revolution; and $\theta_{j}$ is the rotated angle of the spindle when the j−th point is sampled.

$T_{1}$ and $T_{2}$ can also be expressed as

(6) $$\left\{ \begin{array}{l} T_{1}(\theta_{j})=s(\theta_{j})+p(\theta_{j}),j=1,2,\cdots ,n\\ T_{2}(\theta_{j})=s(\theta_{j})-p(\theta_{j}),j=1,2,\cdots ,n \end{array}\right.$$

where s is the radial error of the spindle and p is the roundness error of the standard workpiece.

From Equation (13), s and p can be calculated:

(7) $$\left\{ \begin{array}{l} s(\theta_{j})=\frac{1}{2}(T_{1}(\theta_{j})+T_{2}(\theta_{j})),j=1,2,\cdots ,n\\ p(\theta_{j})=\frac{1}{2}(T_{1}(\theta_{j})-T_{2}(\theta_{j})),j=1,2,\cdots ,n \end{array}\right.$$

## 3. Design of In Situ Measurement and Evaluation System

### 3.1. Overall Structure of In Situ Measurement and Evaluation System

**Figure 6:**
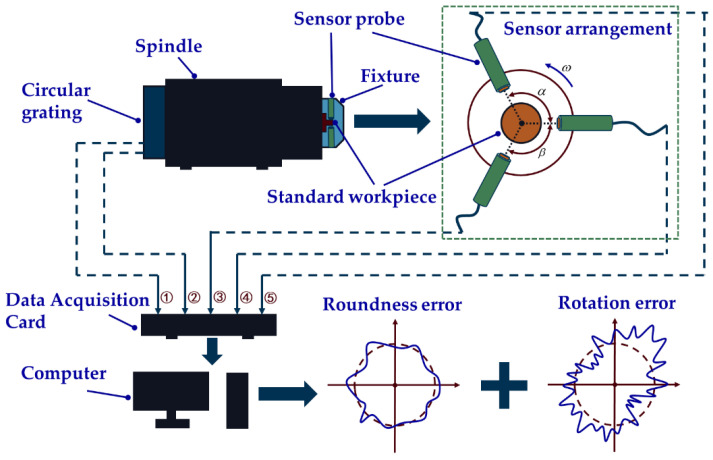
Overall structure of in situ measurement and evaluation system.

Based on the previously theoretical derivations, an in situ measurement and evaluation system is designed, and its overall structure diagram is shown in Figure 6. The system consists of a standard workpiece, fixture, digital capacitive micro-displacement sensors, spindle, circular grating, data acquisition card, computer and servo control system of the machine tool. The reason for choosing capacitive sensors is that compared to other sensors, capacitive sensors are more suitable for working in harsh environments, such as ultra-precision machining workshops. The sensor used in this study is the capacitive micro-displacement sensor designed by the Lion Precision Company. The sensor model is CPL190/C8-2. 0, the range is 5.0 × 10^4^ nm, the resolution is 1.0 nm and the linearity is 0.15% F.S.

The system works as follows: firstly, the standard workpiece, fixture and sensor probes are fixed to the spindle; secondly, a servo control system is used to drive the spindle to rotate at a certain speed, three sensor probes are used to collect data, and two grating data are collected simultaneously; thirdly, the above five signals are imported into the computer and processed. Then, the radial error of the spindle and the roundness error of the standard workpiece can be obtained. Technical details will be given later in the experiments.

### 3.2. Design of Fixture and Standard Workpiece

#### 3.2.1. Design of Fixture

In order to improve the measurement accuracy of the three-point method, an integrated fixture is designed to ensure the accuracy of the sensor installation position. The fixture can be used for both three-point method measurement and Donaldson reversal method measurement.

The problem of harmonic suppression is the main disadvantage of the three-point method measurement, but it can be reduced or even avoided by choosing an appropriate installation angle for the three sensors. In this paper, angle α and β between the three sensors are calculated by referring to the methods from previous research [19,26]. Two sets of sensor arrangements applicable to the three-point method are selected:

(8) $$\left\{ \begin{array}{l} \alpha_{1}=69.984375{}^{\circ}\\ \beta_{1}=70.3125{}^{\circ} \end{array}\right.$$

(9) $$\left\{ \begin{array}{l} \alpha_{2}=146.953125{}^{\circ}\\ \beta_{2}=152.578125{}^{\circ} \end{array}\right.$$

**Figure 7:**
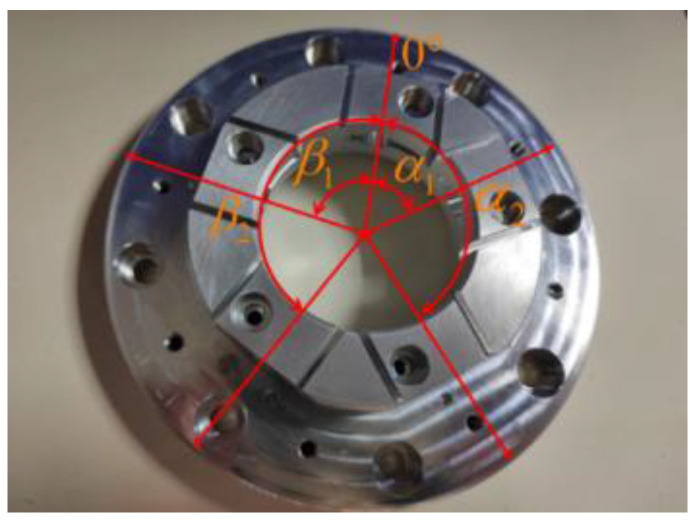
Measuring fixture.

The fixture designed in this paper is shown in Figure 7.

#### 3.2.2. Design of Standard Workpiece

The key to the standard workpiece design is to ensure that the roundness of the standard workpiece is in the same order of magnitude as the radial error of the spindle, so as to ensure the high precision of the error separation.

**Figure 8:**
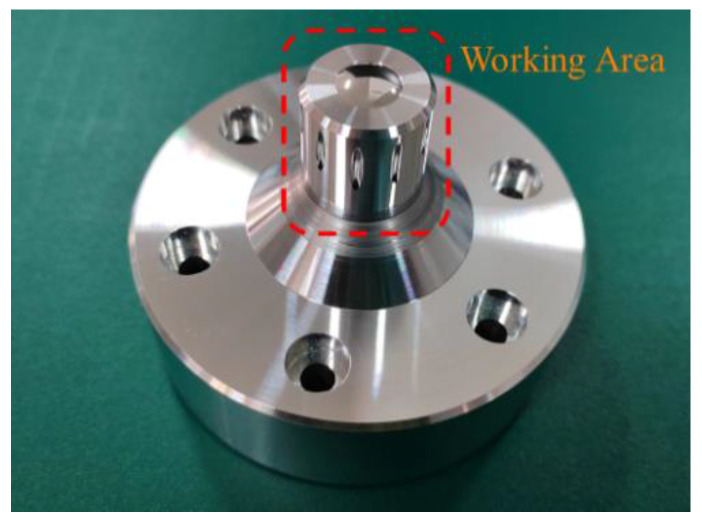
Standard workpiece.

The radial error of the ultra-precision spindle used in this paper is between 10 nm and 50 nm. Therefore, the roundness of the designed standard workpiece should also be guaranteed in the same order of magnitude through ultra-precision turning. The effective working area of the standard workpiece is a cylinder with a diameter of 20 mm, as shown in Figure 8. According to the performance of the ultra-precision lathe, the roundness of the effective working area of the standard workpiece is between 15 nm and 20 nm.

#### 3.2.3. Determination of Sampling Points

The number of sampling points for the three-point method can be determined according to the following algorithm.

The sampling frequency can be expressed by

(10) $$f_{s}=N\frac{\omega_{0}}{2\pi }$$

where N is the number of sampling points per revolution and $\omega_{0}$ is the angular velocity of the spindle.

The frequency component of the signal we want to identify is $f_{\max}$, and the corresponding number of spectral lines is $N_{fm}$. By Nyquist’s sampling theorem, it is required that $f_{s}\geq 2f_{\max}$.

An anti-alias low-pass filter is usually set to filter out the frequency components above $\frac{f_{s}}{2}$. When using a capacitive sensor for measurement, since the capacitive sensor probe pole plate has a certain width, it is equivalent to adding a moving average low-pass filter with a cut-off frequency of

(11) $$f_{c}=\pi \frac{D_{r}}{D}n$$

where $f_{c}$ is the cut-off frequency; $D_{r}$ is the diameter of the standard workpiece; D is the diameter of the capacitive sensor circular pole plate; n is speed of the spindle; and the unit of n is revolutions per minute.

In general, $f_{c}$ will be lower than the cut-off frequency of the anti-alias low-pass filter available, so N has to satisfy

(12) $$f_{s}=N\cdot n\geq 2.56f_{c}$$

Therefore, N can be chosen as

(13) $$N\geq 2.56\pi \frac{D_{r}}{D}$$

The diameter of the sensor pole plate used in this paper is 2 mm, and the diameter of the measured cylindrical workpiece is 20 mm. So according to Equation (13), N can be chosen as 512 points. The filtering effect of the capacitive sensor acts as an anti-aliasing low-pass filter.

## 4. In Situ Measurement and Evaluation Experiment of Radial Error

### 4.1. In Situ Measurement and Evaluation Experiment of Radial Error Using Three-Point Method

The measurement is carried out in the ultra-precision machining workshop. The temperature of the workshop is kept at 20–24 °C, and the humidity is at 50–60%, which mainly changes with the seasons. During the in situ measurement and evaluation experiment, the temperature and humidity are almost constant. The measurement object is an ultra-precision air-bearing spindle independently developed and manufactured by the laboratory. The spindle is installed on an ultra-precision lathe in the machining workshop and the design value of the radial rotation accuracy of the spindle is 20 nm. The main body of the ultra-precision lathe is granite, and the lathe is installed on a shock-absorbing mechanism to achieve a good shock-absorbing effect and ensure the processing performance.

**Figure 9:**
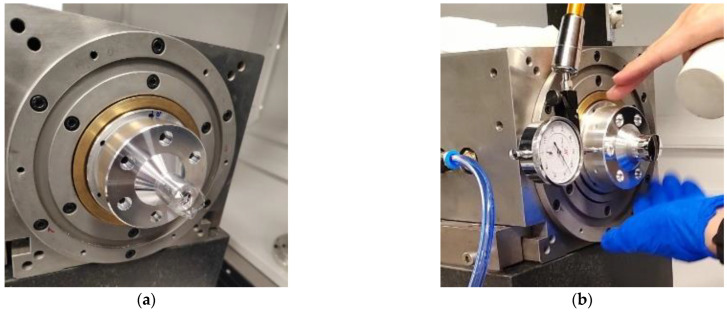

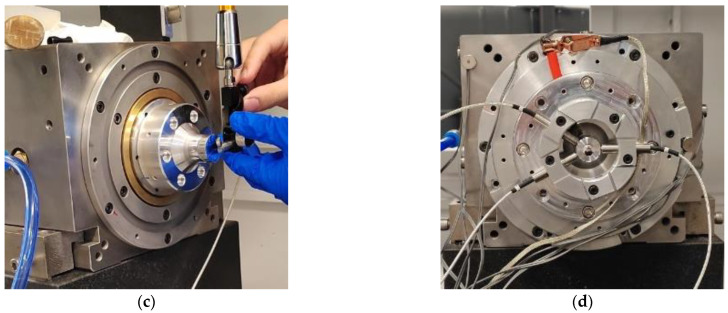
Detailed steps of radial error measurement using three-point method: (**a**) step 1: mounting of standard workpiece, (**b**) step 2: rough adjustment of standard workpiece, (**c**) step 3: fine adjustment of standard workpiece, (**d**) step 4: mounting fixture and sensor probes.

The in situ measurement and evaluation system is used to perform the experiment. The installation position of the three sensors is decided according to Equation (9). Figure 9 shows the detailed measurement process, which is critical to ensure the accuracy and stability of the in situ measurement and evaluation system. Compared with the traditional operation steps of three-point method of measurement, the in situ measurement and evaluation system is more operable while ensuring the installation accuracy.

First, as shown in Figure 9a, the standard workpiece is adsorbed on the air-bearing spindle by the pressure of the vacuum, and the geometric center of the standard workpiece is roughly aligned with the axis of the standard workpiece; second, as shown in Figure 9b, a dial indicator is used to make rough adjustments so that the eccentricity of the standard workpiece is within 1 μm; third, as shown in Figure 9c, the sensor probe is used for fine adjustments to make the eccentricity of the standard workpiece within 0.1–0.2 μm. The more accurate the centering, the higher the accuracy of the measurement results. Finally, the fixture and sensor probes are mounted in place, as shown in Figure 9d, and the measurement can be performed immediately. The measurement results will be presented and analyzed in Section 5.

### 4.2. Comparative Experiment of the Results

The comparative experiment is performed based on the Donaldson reversal method to validate the results measured by the in situ measurement and evaluation system. The spindle analyzer developed by Lion Precision and self-designed experimental equipment are used in the comparative experiment.

**Figure 10:**
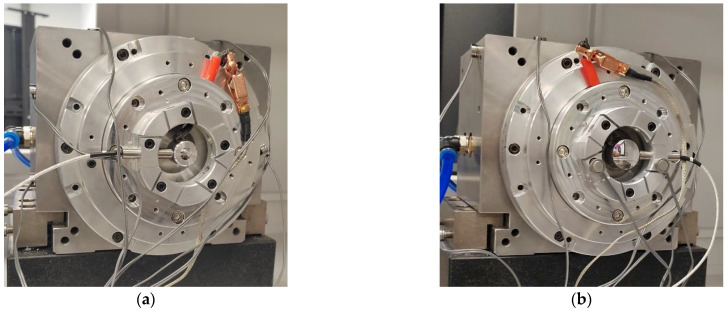
Donaldson reversal method measurement device: (**a**) before reversal, (**b**) after reversal.

The measurement steps of the comparative experiment are as follows: first, the sensor probe is mounted in the 0° direction of the spindle for the first dataset acquisition, as shown in Figure 10a; after this, the fixture, sensor and standard workpiece are reversed 180° at the same time. Then, the sensor probe is in the 180° direction, acquiring the second set of data, as shown in Figure 10b. Before and after reversing, the speed of the spindle should be the same.

**Figure 11:**
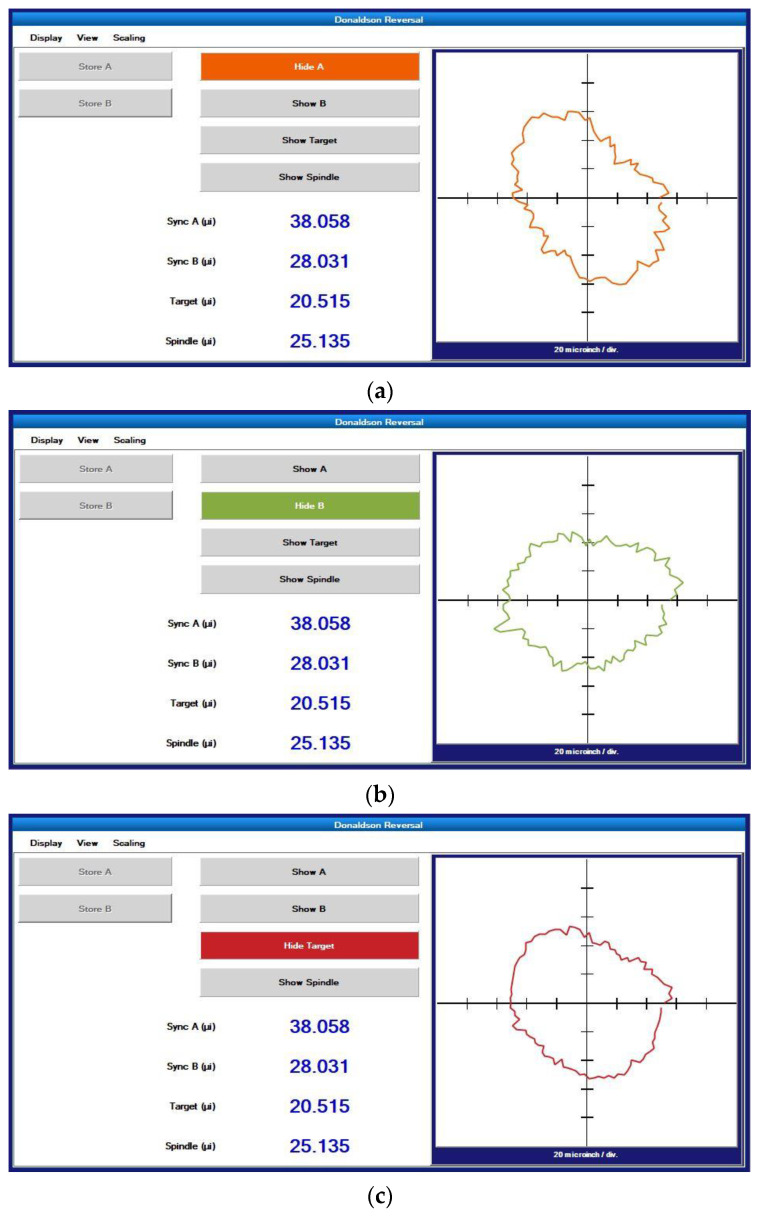

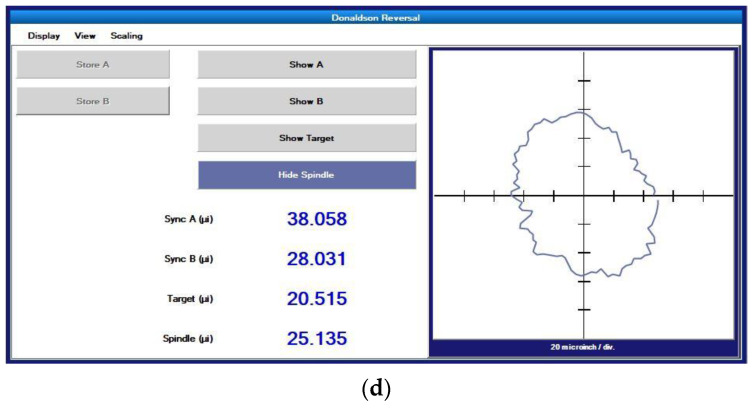
Donaldson reversal method measurement data: (**a**) synchronization error before reversal, (**b**) synchronization error after reversal, (**c**) roundness error of standard workpiece, (**d**) spindle radial error.

The measurement results are shown in Figure 11. The curves in Figure 11a–d show the synchronization error before reversal, the synchronization error after reversal, the roundness error of the standard workpiece and the radial error of the spindle, respectively. The radial error of the spindle is 25.135 nm, and the roundness error of the standard workpiece under testing is 20.515 nm. The measurement results based on the Donaldson reversal method are taken as the reference results. The measurement results obtained using the in situ measurement and evaluation system will be analyzed and discussed together with the reference results in Section 5.

## 5. Result and Discussion

**Figure 12:**
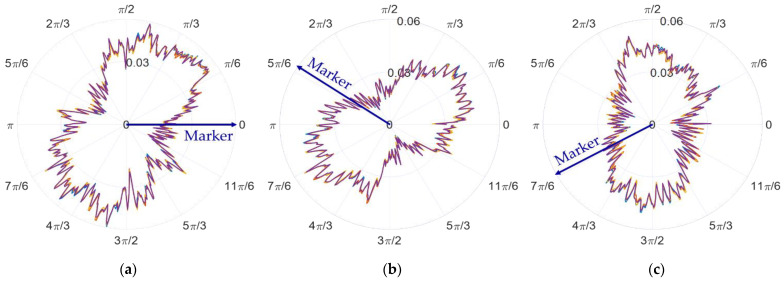
Amplitude phase diagrams of the multi-turn sampling data of the three sensors with the eccentricity and synchronization error removed in the polar coordinate system: (**a**) data from sensor A, (**b**) data from sensor B, and (**c**) data from sensor C.

**Figure 13:**
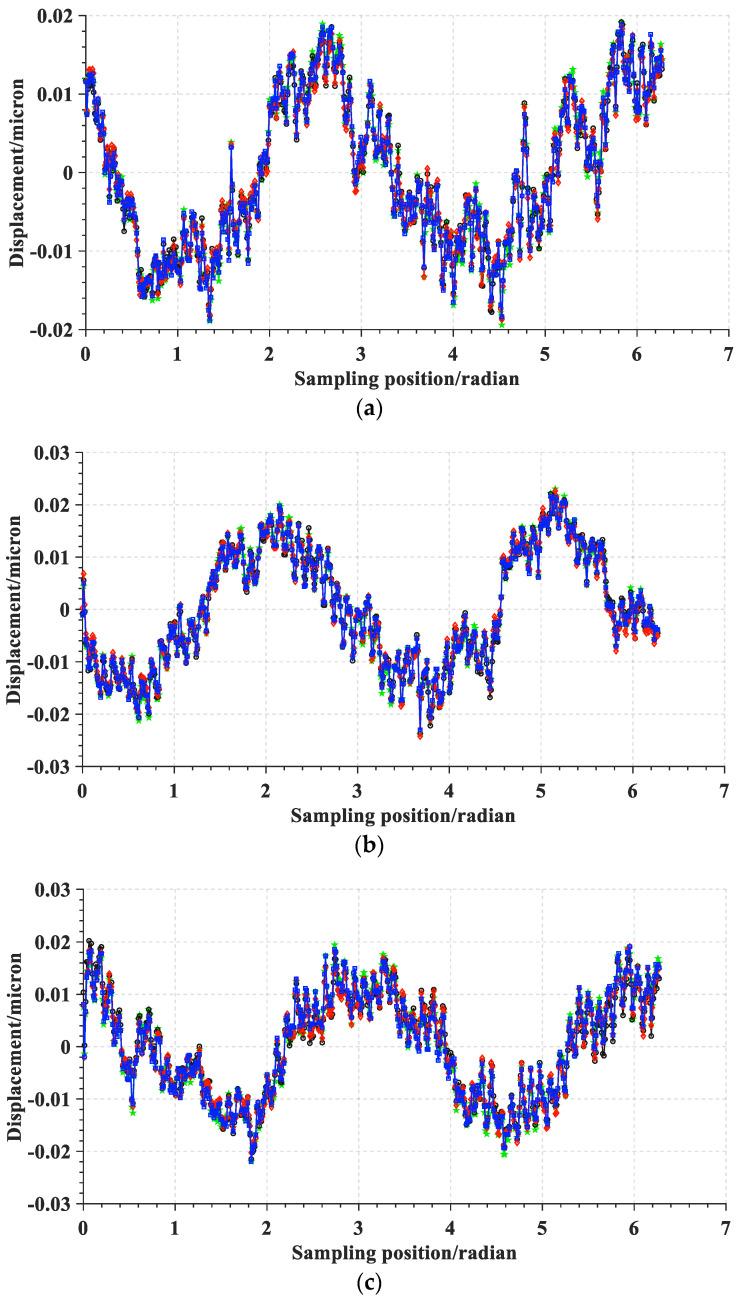
Amplitude phase diagrams of the multi-turn sampling data of the three sensors with the eccentricity and synchronization error removed in the Cartesian coordinate system: (**a**) data from sensor A, (**b**) data from sensor B, and (**c**) data from sensor C.

For the convenience of the discussion, the sampling data when the spindle speed is 60 revolutions per minute is selected as the first example. The raw data obtained by the in situ measurement and evaluation system is processed to remove the asynchronous error and eccentricity. Then, the synchronous error data are obtained. Figure 12a–c shows the amplitude phase diagrams of the multi-turn sampling data of the three sensors with the eccentricity and synchronization error removed in the polar coordinate system, respectively. Figure 13a–c show the amplitude phase diagrams of the multi-turn sampling data of the three sensors with the eccentricity and synchronization error removed in the Cartesian coordinate system, respectively.

It is obvious that the repeatability of the data collected by the sensors is good. By comparing the data in the polar coordinate system, it can be observed that the sampled data of the three sensors show a shape characteristic with a fixed-phase deviation just as the marker in Figure 12 shows. The phase deviation is the same as $\alpha_{2}$ and $\beta_{2}$ determined in Equation (9), reflecting the correctness and stability of the sampling results.

**Figure 14:**
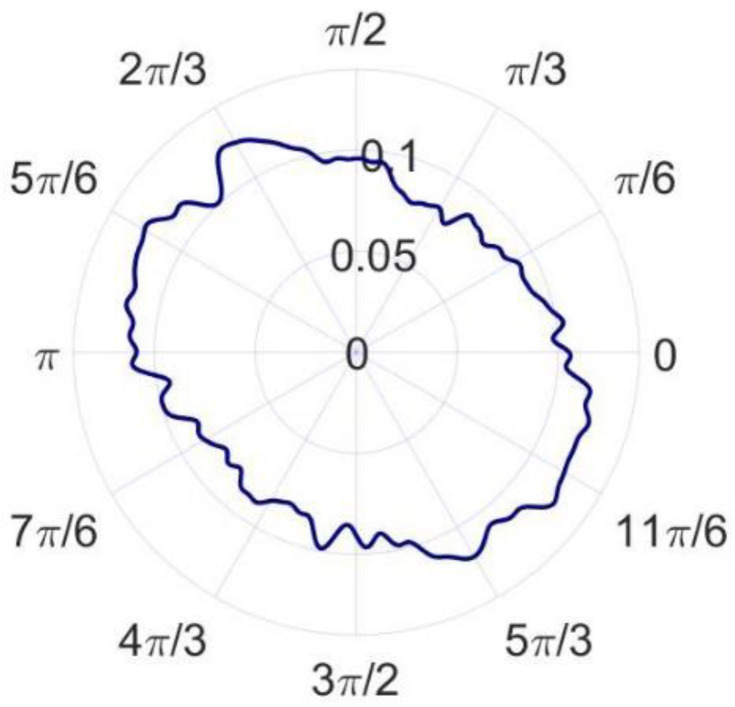
Radial error of the spindle.

**Figure 15:**
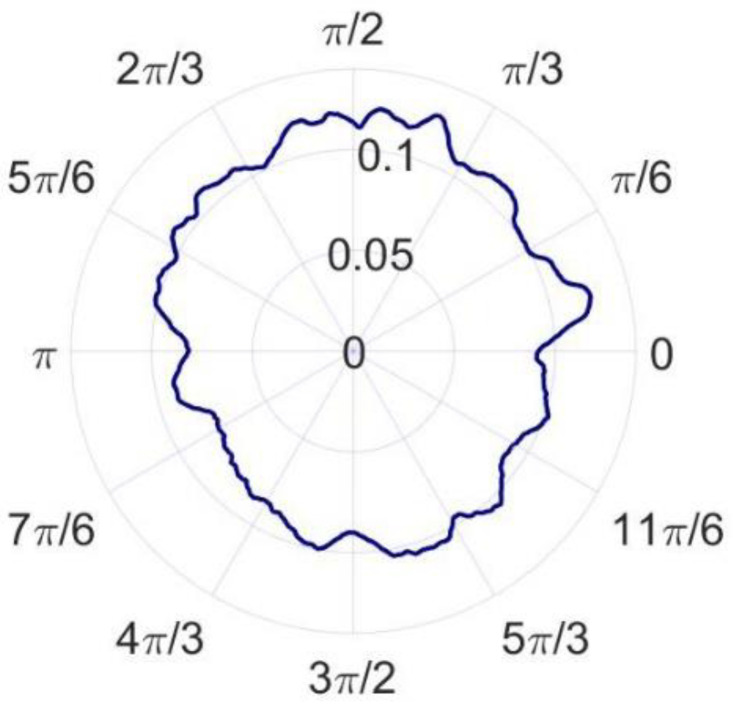
Roundness error of the standard workpiece.

According to the error separation principle of the three-point method, the radial error and roundness error can be calculated, as shown in Figure 14 and Figure 15. The radial error of the spindle and the roundness error of the measured standard workpiece are 22.379 nm and 16.362 nm, respectively. Compared with the reference results, the radial error and roundness error differ from the reference results by 2.756 nm and 4.153 nm, respectively. According to the research results of Eric R. March [17,18], the reversal method has more theoretical advantages than the other methods, but it is also easier to couple more error components. The key to ensuring the measurement accuracy of the reversal method is to minimize the installation angle error and eccentricity error before and after the reversal, so the precision mechanism is generally used for reversal and installation positioning. In the comparative experiment, it is difficult to completely eliminate the error components introduced by the installation angle and eccentricity since no precision mechanism is used. As a result, the roundness error and radial error measured by the reversal method are larger and the measurement results of the in situ measurement and evaluation system are believed to be more accurate.

**Figure 16:**
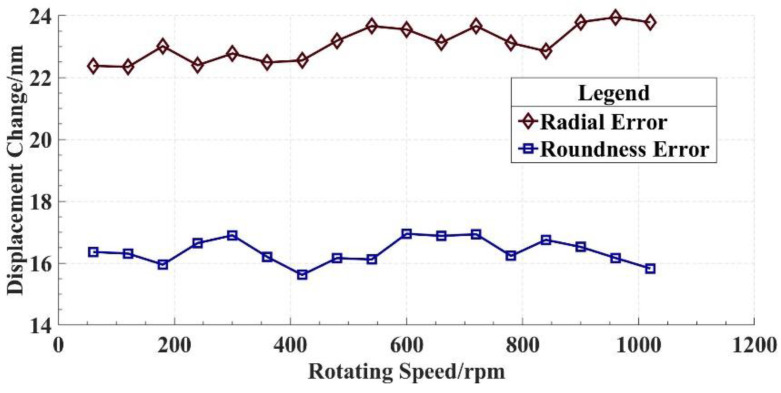
Line graph of measurement results.

In order to validate the reliability and stability of the in situ measurement and evaluation system, measurements based on the system are performed at different spindle speeds. The measurement results are presented in Table 1, and a line graph is drawn based on the measurement results, as shown in Figure 16.

The calculation of the statistical results in Table 1 shows that the average value of the radial error and roundness error are 23.096 nm and 16.386 nm, respectively. The standard deviation of the radial error and roundness error are 0.556 nm and 0.408 nm, respectively. The measurement results of the radial error and roundness error are approximately 2 nm and 4 nm smaller than the reference results, respectively. Considering that no precision mechanism was used for inversion in the comparative experiment, the deviations of the measurement results from the reference results are caused by the secondary clamping error in the comparative experiment. From this point of view, the measurement result of the in situ measurement and evaluation system is more accurate than that of the Donaldson reversal method. Environmental factors such as temperature change and vibration can also lead to the deviations, which can also contribute to the fluctuation in the measurement results shown in Figure 16. The control of environmental factors is extremely important to ensure the radial rotation accuracy of the spindles in actual machining.

Based on the analysis above, it is concluded that the in situ measurement and evaluation system can perform accurate in situ measurements of the radial error in the machining environment. The measurement accuracy of the in situ measurement and evaluation system can reach 20 nm. Since the operation of the system is simpler and fewer clamping errors are introduced, it has advantages over the Donaldson reversal method.

## 6. Conclusions

The three-point method is verified to realize nanometer-scale measurements in the machining environment. An in situ measurement and evaluation system is established and used to perform the experiment. The system simplifies the operation process of the ultra-precision measurement based on the three-point method. In particular, the innovation of the integrated fixture and experimental technical details is the key to improving the in situ measurement accuracy. The accuracy and advantages of in situ measurement and evaluation system are verified through a comparative experiment. The research has a reference function for the practical application of the three-point method.

## Figures and Tables

**Table 1:** Measurement results of radial error by in situ measurement and evaluation system.

Serial Number	Spindle Speed (rpm)	Radial Error (nm)	Roundness Error (nm)
1	60	22.379	16.362
2	120	22.342	16.311
3	180	23.013	15.953
4	240	22.397	16.649
5	300	22.776	16.901
6	360	22.489	16.203
7	420	22.551	15.622
8	480	23.189	16.163
9	540	23.662	16.121
10	600	23.553	16.951
11	660	23.125	16.882
12	720	23.663	16.935
13	780	23.116	16.236
14	840	22.851	16.754
15	900	23.790	16.525
16	960	23.946	16.168
17	1020	23.785	15.828

## Data Availability

The data presented in this study are available upon request from the corresponding author. The data are not publicly available because it is part of an ongoing study.

## References

[1] Yuan J., Lyu B., Hang W., Deng Q. (2017). Review on the progress of ultra-precision machining technologies. Front. Mech. Eng..

[2] Zhang S., To S., Wang S., Zhu Z. (2015). A review of surface roughness generation in ultra-precision machining. Int. J. Mach. Tools Manuf..

[3] Chen G., Sun Y., Zhang F., An C., Chen W., Su H. (2017). Influence of ultra-precision flycutting spindle error on surface frequency domain error formation. Int. J. Adv. Manuf. Technol..

[4] Chen G., Sun Y., An C., Zhang F., Sun Z., Chen W. (2018). Measurement and analysis for frequency domain error of ultra-precision spindle in a flycutting machine tool. Proc. Inst. Mech. Eng. Part B J. Eng. Manuf..

[5] Gao W., Kiyono S., Satoh E., Sata T. (2002). Precision Measurement of Multi-Degree-of-Freedom Spindle Errors Using Two-dimensional Slope Sensors. CIRP Ann..

[6] Geng Z., Tong Z., Jiang X. (2021). Review of geometric error measurement and compensation techniques of ultra-precision machine tools. Light Adv. Manuf..

[7] Aoki Y., Ozono S. (1966). On a New Method of Roundness Measurement Based on the Three-point Method. J. Jpn. Soc. Precis. Eng..

[8] Whitehouse D.J. (1976). Some theoretical aspects of error separation techniques in surface metrology. J. Phys. E Sci. Instrum..

[9] Chetwynd D.G., Siddall G.J. (1976). Improving the accuracy of roundness measurement. J. Phys. E Sci. Instrum..

[10] Donaldson R.R. (1972). A simple method for separating spindle error from test ball roundness error. Ann. CIRP.

[11] Liu X., Rui X., Mi L., Tang Q., Chen H., Xia Y. (2022). Radial Error Motion Measurement and Its Uncertainty Estimation of Ultra Precision Axes of Rotation with Nanometer Level Precision. Micromachines.

[12] Cui H., Lei D., Zhang X., Lan H., Jiang Z., Kong L. (2019). Measurement and analysis of the radial motion error of aerostatic ultra-precision spindle. Measurement.

[13] Cheng H., Hu Q., Xu Y. (2017). Uncertainty evaluation of aerostatic spindle rotation accuracy by donaldson reversal method. Modul. Mach. Tool Autom. Manuf. Tech..

[14] Jozwik J. Dynamic Measurement of Spindle Errors of CNC Machine Tools by Capacitive Sensors During Aircraft Parts Machining. Proceedings of the 2018 5th IEEE International Workshop on Metrology for AeroSpace (MetroAeroSpace).

[15] Ding F., Luo X., Chang W., Wang Z. (2019). In Situ Measurement of Spindle Radial and Tilt Error Motions by Complementary Multi-probe Method. Nanomanufacturing Metrol..

[16] Liu F., Liang L., Xu G., Hou C., Liu D. (2021). Four-Point Method in the Measurement and Separation of Spindle Rotation Error. IEEE/ASME Trans. Mechatron..

[17] Marsh E.R., Arneson D.A., Martin D.L. (2010). A Comparison of reversal and multiprobe error separation. Precis. Eng..

[18] Marsh E., Couey J., Vallance R. (2006). Nanometer-Level Comparison of Three Spindle Error Motion Separation Techniques. J. Manuf. Sci. Eng..

[19] Baek S.-W., Kim M.-G., Lee D.-H., Cho N.-G. (2019). Multi-probe system design for measuring the roundness and rotation error motion of a spindle using an error separation technique. Proc. Inst. Mech. Eng. Part B J. Eng. Manuf..

[20] Tiainen T., Viitala R. (2021). Robust optimization of multi-probe roundness measurement probe angles. Measurement.

[21] Cappa S., Reynaerts D., Al-Bender F. (2014). A sub-nanometre spindle error motion separation technique. Precis. Eng..

[22] Gao W., Kiyono S., Nomura T. (1996). A new multiprobe method of roundness measurements. Precis. Eng..

[23] Gao W., Kiyono S. (1997). On-machine roundness measurement of cylindrical workpieces by the combined three-point method. Measurement.

[24] Ma Y.Z., Wang X.H., Kang Y.H., Dong X. (2013). Roundness Measurement and Error Separation Technique. Appl. Mech. Mater..

[25] Huang R., Pan W., Lu C., Zhang Y., Chen S. (2020). An improved three-point method based on a difference algorithm. Precis. Eng..

[26] Jiao Y., Huang M., Liu P. (2019). Optimal measurement angles of the three-probe spindle error motion separation technique. Meas. Sci. Technol..

